# Advances in Prostate Cancer Treatment: Exploring Molecular Targets and New Strategies in Castration-Resistant Disease

**DOI:** 10.7759/cureus.94427

**Published:** 2025-10-12

**Authors:** Praveen Gopi, Muhammed Ishfaq, Shopon K Das, Zakaria W Shkoukani, Altaf Q Khattak, Hosea B Gana, Rahul Mistry, Richard Jones, Alaa Chamsin, Kaylie E Hughes

**Affiliations:** 1 Department of Urology, Mersey and West Lancashire Teaching Hospitals NHS Trust, Prescot, GBR

**Keywords:** androgen receptor (ar), androgen receptor inhibitor, androgen receptor splice variant 7 (ar-v7), castrate-resistant prostate cancer, parp inhibitors, prostate cancer (pca), radioligand therapy, targeted therapeutics, tumour vaccine

## Abstract

Castration-resistant prostate cancer represents a major clinical challenge, emerging when tumors progress despite standard androgen deprivation therapy. The development of resistance is complex, driven by diverse molecular mechanisms, including androgen receptor (AR) gene amplification and splice variants, as well as AR-independent bypass pathways such as the PI3K/AKT/mTOR and immune checkpoint pathways. These mechanisms involve alternative growth factor signaling, neuroendocrine differentiation, and genetic or epigenetic alterations. This review synthesizes recent advances in understanding these resistance pathways and highlights emerging therapeutic strategies designed to overcome them. Key developments include next-generation AR pathway inhibitors, such as darolutamide, with improved safety profiles; PARP inhibitors for patients with DNA repair gene mutations; and PSMA-targeted radioligand therapy. The therapeutic landscape is also expanding to include novel targets such as the heat shock response, with HSF1 inhibitors like NXP800 in clinical trials for treatment-refractory disease. By targeting this molecular heterogeneity, ongoing research aims to deliver more effective, personalized treatments to improve survival and quality of life for men with advanced prostate cancer.

## Introduction and background

Prostate cancer ranks among the most frequently diagnosed cancers in older men, accounting for 28% of all new cancer cases in males in the United Kingdom [1]. The progression of prostate cancer is heavily dependent on the androgen hormones. Androgens, mainly testosterone, produced in the testes and adrenal glands under luteinizing hormone-releasing hormone and luteinizing hormone (LH) control, are converted to dihydrotestosterone (DHT) by 5‑alpha reductase [2]. The binding of androgens to their specific receptors, the androgen receptors (AR), is crucial for tumor cell proliferation [3]. The AR-DHT complex interacts with specific areas of DNA, and its function as a transcription factor is facilitated by cofactors, resulting in the activation of genes responsive to AR [4]. In healthy prostate tissue, this interaction promotes cellular differentiation, while in cancerous states, it drives uncontrolled cell growth. Tumor-promoting oncogenes often rely on AR-DHT binding and cofactor engagement, indicating therapeutic potential in targeting this pathway [5].

Therapeutic strategies to disrupt the androgen-driven pathway include surgical removal of androgen sources (orchiectomy) or medical suppression of LH production at the pituitary, thereby lowering testosterone output from both the gonads and adrenal glands [6]. Further inhibition is achieved by blocking AR from binding active DHT. Despite achieving low androgen levels, some prostate cancers continue to grow due to persistent AR signaling. This resistance can often be managed by second-line androgen receptor pathway inhibitors (ARPI), such as enzalutamide, apalutamide, and darolutamide, or by using agents like abiraterone acetate that suppress androgen synthesis [7].

Castration-resistant prostate cancer (CRPC) represents a critical stage of prostate cancer that is resistant to traditional androgen deprivation therapies. Multiple mechanisms for CRPC have been identified, involving both androgen-dependent and androgen-independent pathways. However, despite advances in AR pathway inhibition, therapeutic resistance and disease progression remain inevitable in most patients. Current treatments for CRPC often yield limited survival benefits, and predictive biomarkers for therapy selection are not well established. Thus, there exists a critical need to understand the molecular mechanisms underlying resistance and to identify novel therapeutic targets or pathways. A substantial focus in CRPC management is the ongoing search for new molecular targets, aiming to interrupt tumor proliferation by understanding resistance mechanisms and identifying novel therapeutic receptors.

This narrative review aims to synthesize recent advances in the treatment of CRPC, with a focus on emerging molecular targets and innovative therapeutic strategies. The review further explores how combination treatments and novel drug delivery systems are driving personalized medicine, ultimately aiming to improve survival and quality of life in patients with advanced disease.

Background

Androgen Biosynthesis and the Androgen Receptor: Central Role in Prostate Cancer Progression and Therapy

**Figure 1:**
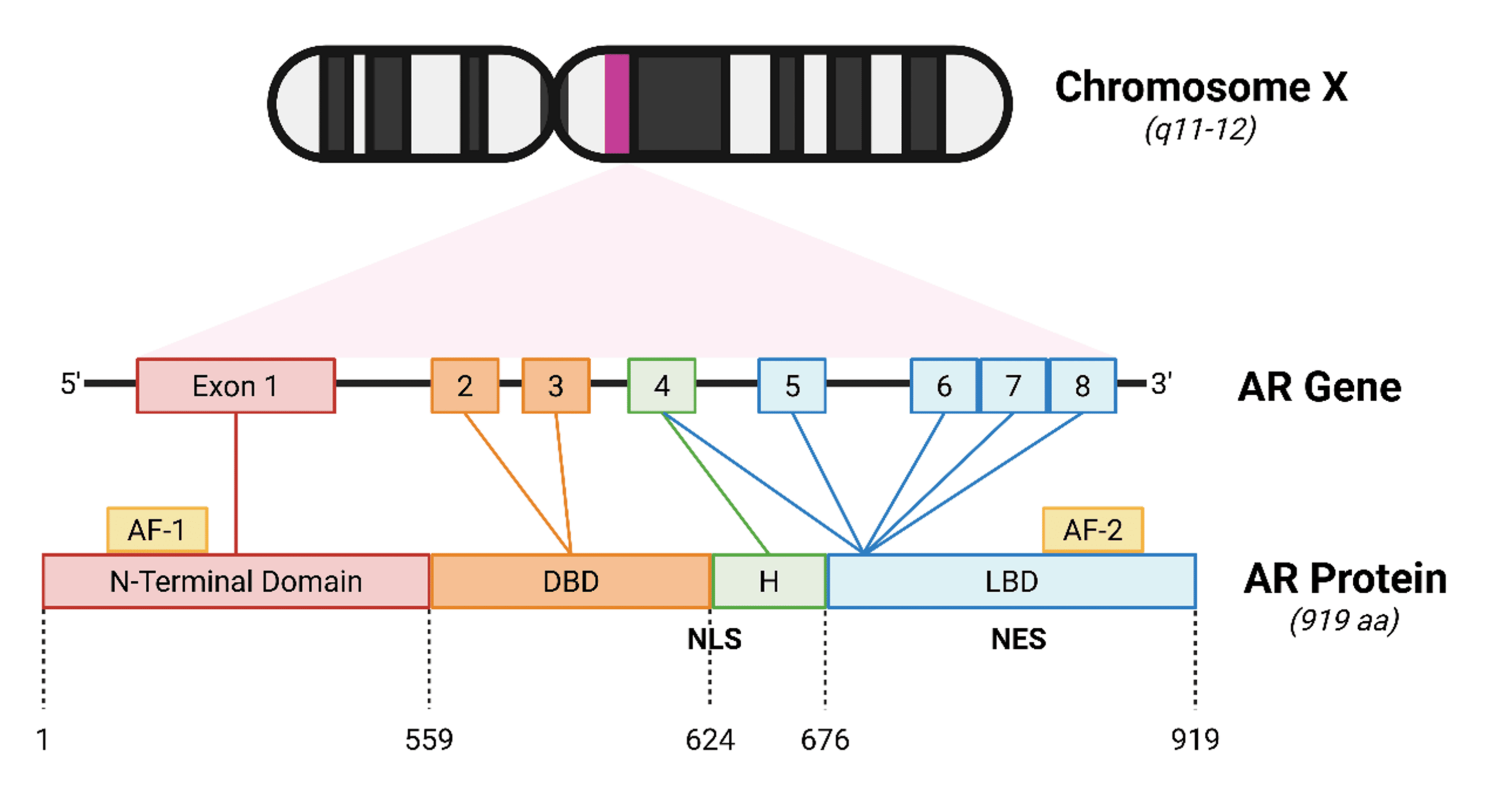
Androgen receptor Created using BioRender.com. AR: androgen receptor, DBD: deoxyribonucleic acid-binding domain, NLS: nuclear localization signal, NES: nuclear export signal, AF: activation factor, H: hinge region

Testosterone is predominantly synthesized by the Leydig cells in the testes, with a smaller proportion derived from the adrenal glands. Its production is governed by LH, which is secreted by the anterior pituitary, stimulating key enzymatic pathways involved in steroidogenesis [8]. Following its release into the circulation, testosterone exerts its effects either directly or by being converted into the more potent metabolite DHT in peripheral tissues. The physiological actions of both testosterone and DHT are mediated via the AR. The AR is a nuclear hormone receptor that plays a pivotal role in the development and progression of prostate cancer (Figure 1). It is encoded by the AR gene, located on the X chromosome at position Xq11-12. It translates into a ligand-activated transcription factor that mediates the biological effects of androgens such as testosterone and DHT [8,9]. Structurally, the AR protein consists of key functional domains, including an N-terminal transactivation domain, a central DNA-binding domain, and a C-terminal ligand-binding domain. Upon binding to its ligand, the receptor undergoes conformational changes, translocates to the nucleus, and binds to androgen response elements within the promoters of target genes, initiating the transcription of genes involved in prostate tissue growth, survival, and differentiation [10].

Clinically, the AR remains a central therapeutic focus in CRPC. Despite effective testosterone suppression through androgen deprivation therapy (ADT), many tumors continue to rely on AR signaling for progression. This continued activity may result from AR gene amplification, point mutations, increased AR expression, or the emergence of AR splice variants such as AR-V7, which can activate transcription in the absence of ligand binding [9]. As a result, therapies targeting the AR pathway, such as next-generation AR antagonists and agents that inhibit androgen biosynthesis, remain the cornerstone of CRPC management, emphasizing the clinical importance of AR in the resistance and evolution of advanced prostate cancer.

Androgen Deprivation Therapy in Prostate Cancer

Although ADT is usually effective at first, prostate cancer almost always adapts and progresses to a state known as CRPC [8,10]. This resistance typically develops within 18 to 36 months after starting hormonal therapy, particularly in patients with metastatic disease [11]. One of the key limitations of ADT is its inability to entirely suppress all sources of testosterone, especially androgens produced by the adrenal glands and within tumor tissue [12]. In addition, prostate cancer cells develop mechanisms to survive in low-testosterone environments by increasing AR expression, acquiring AR gene amplifications, or producing AR splice variants that function independently of androgens [9]. Moreover, long-term ADT is associated with a range of adverse effects, including reduced libido, erectile dysfunction, osteoporosis, insulin resistance, and cardiovascular risk, significantly impacting the quality of life [13]. In response to these challenges, novel therapies and combination treatment strategies are being explored to prolong disease control and delay the onset of resistance in CRPC [14].

Mechanisms of resistance in prostate cancer

Androgen Receptor-Dependent Mechanisms

**Figure 2:**
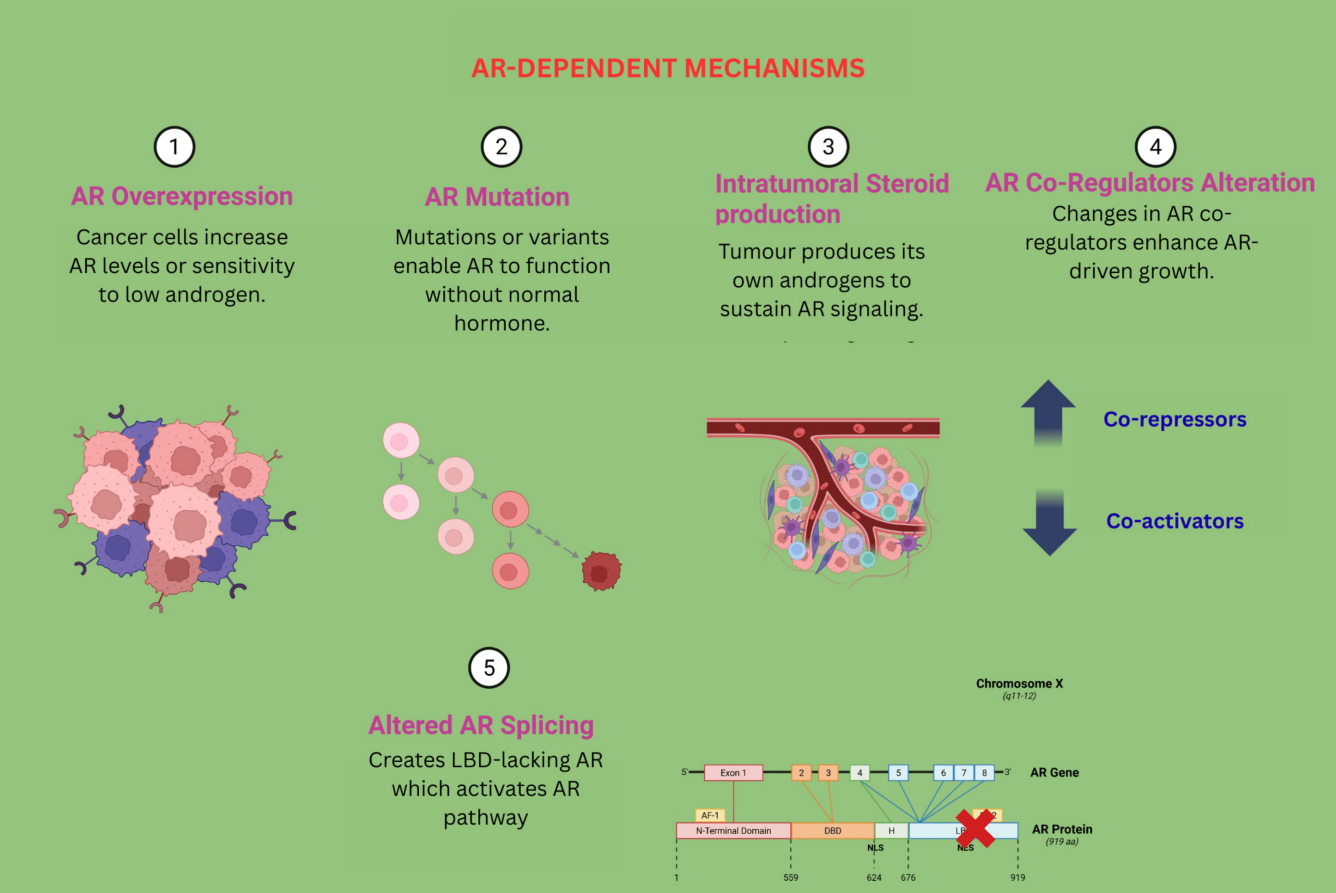
AR-dependent mechanisms of CRPC showing where CRPC maintains AR pathway activation despite androgen deprivation Created using BioRender.com. CRPC: castrate-resistant prostate cancer, AR: androgen receptor, LBD: ligand binding domain

AR-dependent mechanisms are central to the pathogenesis and progression of CRPC, allowing tumor cells to maintain growth and survival despite systemic androgen deprivation (Figure 2). One of the most frequently observed mechanisms is AR gene amplification and overexpression, resulting in increased levels of AR protein within cancer cells. This amplification renders the tumor hypersensitive to the low androgen concentrations present after medical or surgical castration, enabling continued AR signaling and disease progression. AR amplification is found in approximately 20-80% of CRPC cases, compared to its rarity in treatment-naive prostate cancer [15].

AR mutations, particularly within the ligand-binding domain, represent another vital resistance mechanism. These mutations expand the receptor’s ligand specificity, allowing it to be aberrantly activated by non-androgenic steroids such as glucocorticoids or progesterone, and in some cases, convert AR antagonists into agonists. This form of "promiscuous" AR activation undermines the efficacy of ADT, contributing to the persistence of tumor growth [9]. Another critical mechanism involves AR splice variants, such as AR-V7, which lack the ligand-binding domain altogether but remain transcriptionally active. These constitutively active AR isoforms drive cancer cell proliferation in the absence of circulating androgens or ligand stimulation. They are frequently associated with resistance to AR-targeted therapies, such as enzalutamide or abiraterone [16]. Moreover, intratumoral androgen synthesis has been identified as a key adaptive response. Tumor cells upregulate steroidogenic enzymes that allow the de novo production of androgens from cholesterol or adrenal precursors, effectively bypassing systemic blockade. This locally synthesized androgen is sufficient to activate the AR signaling pathway, further promoting tumor growth [15]. Lastly, changes in AR coregulators, including coactivators and corepressors, represent an additional layer of resistance. Alterations in the expression or function of these transcriptional regulators enhance or modify AR activity, enabling sustained gene transcription even under low-androgen conditions [9]. These modifications to AR-interacting proteins contribute to therapy resistance and reinforce the functional integrity of AR signaling in CRPC.

Collectively, these AR-dependent mechanisms enable prostate cancer cells to resist androgen deprivation and continue to proliferate, highlighting the complexity of CRPC biology and the need for advanced therapeutic strategies that can effectively overcome or bypass AR signaling pathways.

Androgen Receptor-Independent (Bypass) Mechanisms

**Figure 3:**
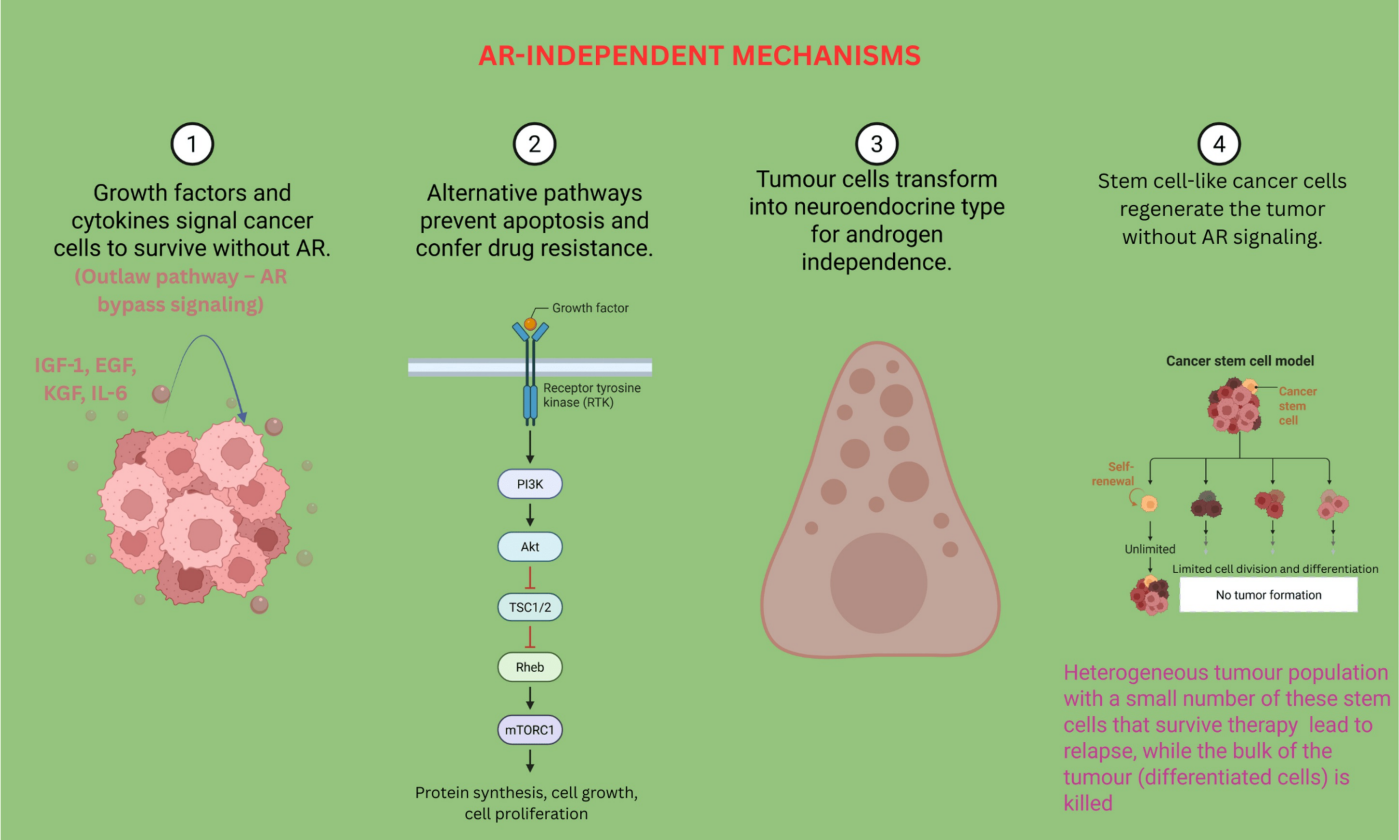
AR-independent mechanism of CRPC showing resistance pathways whereby prostate cancer cell survival and proliferation occur independent of AR signaling Created using BioRender.com. CRPC: castrate-resistant prostate cancer, AR: androgen receptor, LBD: ligand binding domain

In CRPC, tumor cells can continue to grow even when AR signaling is blocked with the help of AR-independent pathways (Figure 3). These mechanisms enable cancer cells to bypass the need for androgen hormones and continue growing in environments where these hormones are significantly reduced due to treatment.

One key mechanism involves the activation of alternative growth and survival pathways. Even without the presence of androgens, cancer cells can still receive growth signals from other molecules, such as IGF-1, EGF, and KGF, as well as from receptor systems like HER-2/neu. These molecules activate internal signaling networks, helping cancer cells continue to divide and survive [14,17]. Inflammatory messengers, such as IL-6, can also help cancer cells resist therapy by activating survival pathways outside of the AR signaling pathway. Another primary strategy involves the upregulation of survival-promoting proteins, such as Bcl-2, and the activation of internal biochemical pathways like PI3K/Akt and Src kinases. These pathways make the tumor cells more resistant to programmed cell death (apoptosis), allowing them to survive longer and become harder to eliminate, even without AR involvement [18,19]. CRPC also becomes more challenging to treat when tumors shift into a more aggressive form known as neuroendocrine prostate cancer (NEPC). In this state, cancer cells acquire features similar to those of nerve cells and produce substances such as serotonin and bombesin, which promote tumor growth in a hormone-independent manner. These neuroendocrine-like tumors typically lack AR expression and, as a result, do not respond to hormone-based therapies [20]. Lastly, recent studies have shown that prostate cancer stem cells, a rare population of cells within the tumor, can survive without needing AR signaling. These stem-like cells have the unique ability to self-renew and regenerate the tumor over time, making them inherently resistant to androgen deprivation and likely contributors to disease recurrence [21,22].

Together, these AR-independent or bypass mechanisms illustrate the complexity of CRPC and highlight the need for treatment strategies that extend beyond targeting AR signaling. By focusing on these alternative pathways, future therapies may more effectively address treatment resistance and disease progression in patients with advanced prostate cancer.

Genetic and Epigenetic Alterations in Castration-Resistant Prostate Cancer

**Figure 4:**
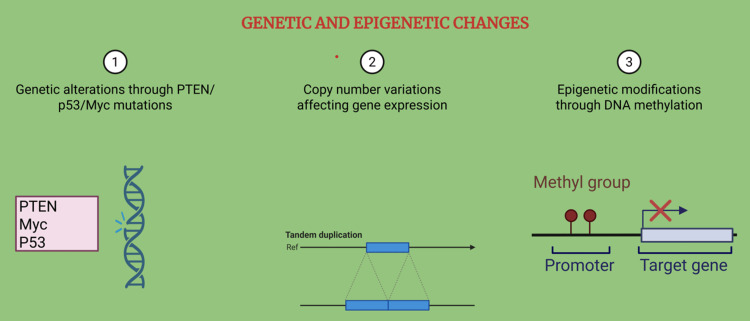
Genetic and epigenetic changes in CRPC Created using BioRender.com. CRPC: castrate-resistant prostate cancer

In addition to changes in AR signaling, genetic and epigenetic alterations also play a significant role in the development and progression of CRPC (Figure 4). These changes can either work in conjunction with AR-dependent processes or act independently, allowing prostate cancer cells to grow even when androgen levels are very low due to treatment.

One key genetic mechanism involves mutations in critical cancer-related genes, including tumor suppressors such as TP53 and PTEN, as well as oncogenes like Myc. For example, loss of PTEN leads to overactivation of the PI3K/Akt pathway, which helps cancer cells survive without hormone signaling. Similarly, amplification of the Myc gene promotes uncontrolled cell growth. These mutations disrupt the normal regulation of the cell cycle and DNA repair, helping prostate cancer cells resist therapy and continue to spread [23,24].

In addition to these genetic mutations, epigenetic changes, which are chemical modifications to DNA and the proteins that package DNA, can also contribute to castration resistance. These include DNA methylation, histone modifications, and changes in chromatin structure, all of which can alter gene expression without changing the actual DNA sequence. These modifications may switch off genes that suppress tumors or activate genes that encourage cancer cell survival and growth, even in the absence of androgens [25].

Together, these genetic mutations and epigenetic alterations help prostate cancer cells adapt to treatment by reprogramming their internal machinery, ultimately allowing them to resist hormone therapy and continue progressing. Understanding these changes is crucial for developing new, targeted treatments that go beyond hormone-based approaches.

## Review

Newer receptor targets

Prostate-Specific Membrane Antigen

**Figure 5:**
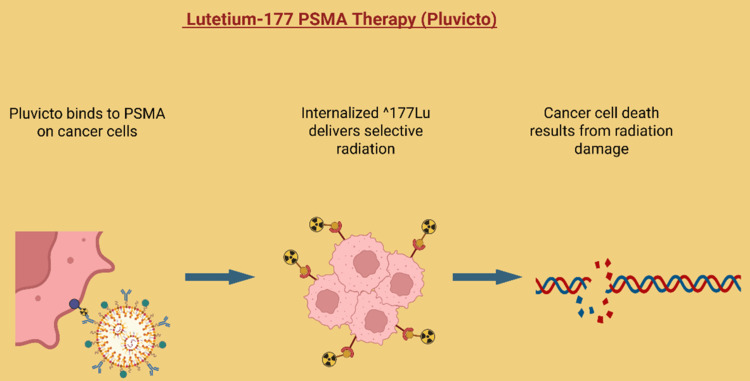
177Lu-PSMA radioligand therapy. Pluvicto targets and binds to PSMA receptors (overexpressed on prostate cancer cells) and is internalized to deliver localized beta radiation from the 177Lu isotope, which causes DNA damage and ultimately leads to cancer cell death Created using BioRender.com. PSMA: prostate-specific membrane antigen, 177Lu: lutetium, DNA: deoxyribonucleic acid

Prostate-specific membrane antigen (PSMA) is highly expressed in advanced CRPC and serves as a target for diagnostic imaging and therapy. Lutetium-177 (177Lu) PSMA, a radioligand therapy, is now approved for metastatic CRPC (mCRPC) that has progressed after standard treatments [26]. 177Lu PSMA therapy, marketed as Pluvicto, targets the PSMA found on prostate cancer cells [27]. This therapy combines a small molecule that specifically binds to PSMA with the radioactive isotope 177Lu, which delivers targeted radiation directly to cancer cells while sparing most healthy tissue (Figure 5). Pluvicto has received approval from both the Food and Drug Administration (FDA) and the European Medicines Agency (EMA) for use in patients with PSMA-positive mCRPC, particularly those whose disease has progressed despite ARPI therapy. It has also recently been approved for use even before chemotherapy is required [28]. The availability of Pluvicto is expanding in specialized centers with nuclear medicine capabilities across the United States, the United Kingdom, and Europe, providing an important option for patients with advanced, treatment-resistant disease [27]. Key clinical trials, including the VISION and PSMAfore studies, have demonstrated that 177Lu PSMA therapy can significantly improve radiographic progression-free survival, overall response rates, and quality of life while maintaining manageable side effects. By precisely targeting PSMA-expressing cancer cells, this therapy offers new hope for patients whose cancer is no longer responding to standard hormone treatments. It can help delay the need for chemotherapy [26,29].

Heat Shock Proteins

Heat shock proteins (HSPs and HSF1) assist tumor survival under stress and resistance conditions [30]. NXP800 and other HSF1 inhibitors are in clinical trials targeting diseases refractory to conventional options [31]. NXP800 is an investigational anticancer agent that exerts its effects by inhibiting the HSF1 signaling pathway, a crucial regulator of the cellular stress response in cancer cells [32]. By disrupting HSF1 activity, NXP800 induces an unfolded protein response, leading to proteotoxic stress, suppression of AR activity, and ultimately, inhibition of tumor growth [33]. While NXP800 has not yet been approved for the treatment of prostate cancer, it has received FDA Fast-Track and Orphan Drug Designation for certain other solid tumors, such as ARID1a-deficient ovarian cancer and cholangiocarcinoma, reflecting its potential in addressing unmet medical needs [34]. Ongoing early-phase clinical trials are evaluating NXP800 in patients with advanced and enzalutamide-resistant prostate cancer, where resistance to currently available ARPIs presents a major therapeutic challenge [35]. Preclinical studies, including laboratory and animal models, have demonstrated robust antitumor effects of NXP800, even in hormone-resistant CRPC, providing strong scientific support for its continued development [33]. However, at present, NXP800 remains an experimental therapy. It is only accessible within the context of clinical trials, necessitating further research to determine its safety, efficacy, and future role in the management of treatment-resistant prostate cancer [31].

Estrogen Receptors

Estrogen receptors (ERs) play a dual role in prostate cancer, with ERα supporting proliferation and ERβ potentially offering tumor-suppressive effects. Selective ER modulators and antagonists are under investigation; however, no agents are currently approved specifically for CRPC that target these pathways [36]. In CRPC, intratumor androgen and estrogen (E2) synthesis both increase, partly due to elevated aromatase activity driven by the activation of alternative promoters of the CYP19A1 gene. These promoters, normally inactive in healthy prostate cells, become active during disease progression, enhancing aromatase expression and contributing to hormone-driven tumor growth. ERs, particularly ERα and ERβ, play essential but opposing roles in the development and progression of CRPC [37]. While ERα promotes proliferation and inflammation of prostate cancer cells, driving disease progression even after ADT, ERβ generally exhibits antiproliferative and pro-apoptotic effects that suppress tumor growth [38]. The loss or reduction of ERβ, alongside continued activation of ERα, contributes to the shift towards CRPC [37]. Recent therapeutic strategies are targeting these receptors. Selective estrogen receptor modulators (SERMs), such as toremifene and raloxifene, as well as selective estrogen receptor downregulators (SERDs) like fulvestrant, have been explored in clinical settings [39]. Additionally, advances in nanotechnology have enabled the targeted delivery of ERα antagonists (e.g., nano-formulated toremifene), while ERβ-selective agonists are under investigation to enhance the anticancer properties of ERβ [37]. Overall, novel ER-targeted therapies represent a promising avenue for overcoming resistance in advanced prostate cancer [37].

STEAP1

STEAP1, a six-transmembrane epithelial antigen of the prostate, is highly overexpressed in CRPC and shows minimal expression in normal tissues, making it an attractive therapeutic target [40]. It contributes to tumor progression by promoting cancer cell proliferation, invasion, and metastasis. Several innovative treatments are under development, including antibody-drug conjugates (ADCs), CAR T-cell therapies, T-cell engagers, and STEAP1-based vaccines [41]. These approaches aim to harness the immune system and targeted drug delivery to specifically attack STEAP1-expressing cancer cells, potentially overcoming resistance in CRPC [40].

Somatostatin/Neurotensin Receptors

Somatostatin and neurotensin (NTS) are involved in the progression of CRPC and represent potential therapeutic targets. NTS, produced by neuroendocrine cells, may enhance the invasive properties of prostate cancer cells [42]. In contrast, somatostatin and its synthetic analogues can suppress hormone secretion and have demonstrated therapeutic potential in managing CRPC, especially in cases exhibiting neuroendocrine differentiation [43].

Lanreotide, a long-acting somatostatin analogue, has shown promise in treating CRPC by targeting tumor-promoting secretions in the tumor microenvironment. Encouraging clinical responses and quality-of-life improvements warrant further investigation in large-scale trials [44]. Somatostatin receptor (SSTR) imaging and theranostics are now essential methods for diagnosing and treating neuroendocrine tumors (NENs). They work by targeting the high levels of certain SSTR types, mainly types 2, 3, and 5, which these tumors often have. The combination of SSTR PET imaging and peptide receptor radionuclide therapy, particularly using agents like 177Lu-DOTATATE, has significantly improved outcomes in well-differentiated NENs. SSTR PET also helps predict therapy response and assess disease heterogeneity, although its role in treatment monitoring requires further validation [45]. Recent research has highlighted that targeting NTS signaling, particularly through the inhibition of the NTSR1 receptor, can delay neuroendocrine differentiation and enhance the effectiveness of AR-targeted therapy in CRPC. Combining an NTSR1 antagonist with AR antagonists like enzalutamide may present a promising therapeutic strategy to prevent or slow the onset of neuroendocrine disease and CRPC [46].

Glucocorticoid Receptors

Recent research indicates that the glucocorticoid receptor (GR) plays a pivotal role in promoting resistance to AR-targeted therapies in CRPC, thereby significantly influencing treatment outcomes [47]. Efforts are ongoing to develop selective GR modulators or antagonists, such as RU-486, as blocking the GR pathway may help overcome resistance and improve patient response to current therapies [48]. Investigations are also examining how GR interacts with other transcription factors, aiming to understand and disrupt the mechanisms that drive therapy resistance in CRPC.

Integrins and Filamin A

Integrins play a key role in the treatment and progression of CRPC by mediating cancer cell adhesion, migration, and survival signaling. Specific integrins, such as αvβ6 and α6β1, promote CRPC growth by activating pathways that enhance AR activity even in low-androgen conditions, contributing to therapy resistance and tumor aggressiveness [49]. Examples include monoclonal antibodies like abituzumab, which targets αv integrins, and small molecules such as cilengitide, which inhibits αvβ3 and αvβ5 integrins. Both show potential in reducing tumor growth and metastatic spread in CRPC [50].

Filamin A (FlnA) is an actin-binding protein that regulates cytoskeletal organization and cellular signaling, playing a critical role in the progression of CRPC [51]. Tumor-suppressive effects are linked to its nuclear localization, where it inhibits cell migration and enhances sensitivity to ADT [52]. In contrast, cytoplasmic retention of FlnA promotes cancer cell motility, metastasis, and resistance to hormone therapy [53]. Therapeutic strategies that induce FlnA nuclear translocation, such as enhancing calpain-mediated cleavage or blocking FlnA interaction with the AR, have shown promise in restoring treatment sensitivity and inhibiting CRPC progression [51,53].

Chromatin Coactivators of Androgen Receptor Signaling

CBP and p300 are key chromatin coactivators and histone acetyltransferases that play a critical role in CRPC by enhancing AR signaling and promoting tumor growth. Their elevated expression in CRPC is associated with poor prognosis and resistance to ADT, as they maintain AR transcriptional activity even in low-androgen conditions. Inhibiting CBP/p300, particularly through targeting their bromodomains, disrupts AR-driven gene expression and impairs DNA damage repair mechanisms, resulting in reduced cancer cell proliferation and sensitization to standard therapies [54]. GNE-049 is a potent and selective inhibitor targeting the CBP/p300 bromodomain, developed by Genentech, and has shown promising results in early laboratory studies using prostate cancer models [55]. Another example is CCS1477, a newer small-molecule inhibitor that also blocks the p300/CBP bromodomain and has demonstrated the ability to reduce tumor growth in AR-driven and mCRPC models [56]. These CBP/p300 inhibitors may be most effective when combined with other treatments, such as AR-targeted therapies or drugs that affect DNA damage repair, to enhance their efficacy [57]. Currently, several of these inhibitors are being tested in clinical trials for different types of cancer, including prostate cancer, to evaluate their safety and effectiveness in patients [58].

NXTAR

NXTAR is a long non-coding RNA that plays a key tumor-suppressor role in CRPC by lowering the expression of the AR and its variant AR-V7 [59]. It is located close to the AR gene and acts by binding upstream of the AR promoter, recruiting the EZH2 enzyme to place repressive epigenetic marks, thereby silencing AR signaling [60]. Since continued AR activity is a primary factor in CRPC progression and treatment resistance, especially to drugs like enzalutamide, restoring NXTAR expression through genetic methods or small molecules like the ACK1/TNK2 inhibitor (R)-9b has shown success in reducing AR/AR-V7 levels and shrinking drug-resistant prostate tumors in preclinical studies [59]. Furthermore, synthetic RNA molecules based on NXTAR, such as NXTAR-N5, have been found to mimic its effects, offering a promising therapeutic strategy for advanced and treatment-resistant prostate cancer [59].

Liver X Receptors

Liver X receptors (LXRs), known as LXRα and LXRβ, are becoming promising targets for treating CRPC due to their essential role in controlling cholesterol and influencing how cancer cells grow and behave [60,61]. These receptors function as sensors for cholesterol and related molecules, helping to maintain the activity of genes involved in fat metabolism, inflammation, and immune responses, all of which are linked to cancer development [62,63]. When LXRs are activated, they can stop cancer cells from multiplying, help trigger cancer cell death and cleanup processes like autophagy, and make tumors shrink by disrupting key growth signals in cancer [64]. Studies in the lab have shown that LXR-activating drugs can slow down CRPC and boost the body's immune response to tumors. At the same time, loss of LXR function, often caused by increased levels of a gene called SULT2B1b, can lead to more aggressive cancer by increasing enzymes like AKR1C3 and activating survival pathways like ERK1/2 [6,65,66]. Researchers are now studying selective LXR modulators as potential treatments to be used in conjunction with current therapies. Still, their success can depend on the specific type of cancer and the tumor environment, which can influence how well LXRs work to suppress tumor growth [63,67].

T-LAK Cell-Originated Protein Kinase

T-LAK cell-originated protein kinase (TOPK) plays a critical role in CRPC by promoting AR signaling, especially the AR-V7 variant, which enables cancer cells to grow without androgens [68]. Elevated TOPK expression also supports cell migration, invasion, and survival by activating cancer-promoting pathways, including PI3K/AKT and ERK. It is associated with more aggressive and therapy-resistant disease [69]. Inhibiting TOPK can suppress AR signaling, reduce tumor growth, and decrease metastatic potential, making it a valuable target for therapy in CRPC [70]. Pharmacologic inhibitors of TOPK (such as OTS-514) suppress AR signaling and tumor cell proliferation, suggesting their potential in CRPC therapy [71]. Because TOPK is highly expressed in tumor tissues but largely absent from normal adult tissues, it is also being explored as a potential biomarker for advanced and resistant prostate cancer. Overall, targeting TOPK could offer novel therapeutic opportunities for managing CRPC and overcoming resistance to current treatments [72].

Pathway targeted therapy

Androgen Receptor Pathway Inhibitors

**Figure 6:**
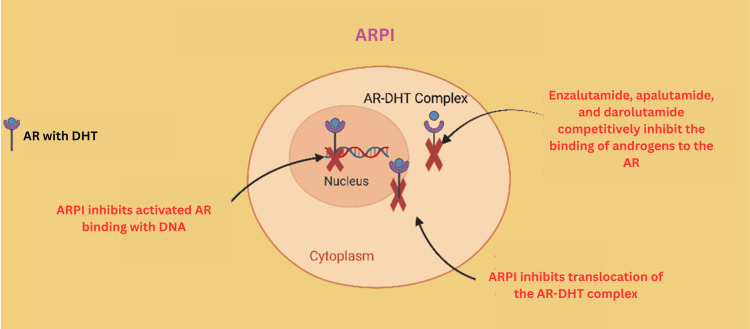
ARPI mechanism of action Created using BioRender.com. ARPI: androgen receptor pathway inhibitor, AR: androgen receptor, DHT: dihydrotestosterone

ARPIs like enzalutamide, apalutamide, and darolutamide have transformed the management of advanced prostate cancer. These medications target the AR signaling that drives prostate cancer growth [73] (Figure 6). ARPIs are now established as standard treatments for both hormone-sensitive and CRPC, offering benefits whether used alone or alongside traditional hormone therapies [74].

Enzalutamide is a powerful ARPI that blocks androgen binding and the actions of the AR inside the cell nucleus [75]. It is widely approved and used for both non-metastatic and metastatic forms of CRPC, as well as for patients with high-risk, earlier-stage disease. Enzalutamide has been proven, in clinical trials such as PROSPER, to significantly extend the time before cancer spreads, improve overall survival, and delay disease progression [76].

Apalutamide, another second-generation ARPI, works by preventing AR activation and movement into the cell nucleus. It is approved for men whose prostate cancer remains sensitive to hormones but who are at high risk of developing metastases, as well as for metastatic hormone-sensitive prostate cancer (mHSPC) [73]. In the SPARTAN trial, apalutamide significantly prolonged metastasis-free survival, achieving a median of 40.5 months compared to 16.2 months for placebo, with a hazard ratio for metastasis or death of 0.28 [77]. The trial also showed improvement in overall survival and time to symptomatic progression for men at high risk [78].

Darolutamide is notable for its unique molecular structure, which significantly limits its ability to penetrate the blood-brain barrier. This property reduces the risk of central nervous system side effects compared to other ARPIs [79]. Darolutamide works by strongly inhibiting the AR and is approved for non-mCRPC and, in combination with hormone therapy and chemotherapy, for mHSPC. In the ARAMIS trial, darolutamide significantly improved both metastasis-free and overall survival; the benefit was observed even among patients with multiple comorbidities and those taking other medications [80]. The ARASENS trial confirmed increased survival when darolutamide was added to standard hormone therapy and chemotherapy for patients with mHSPC, with about 63% of darolutamide-treated patients alive at four years versus 50% for placebo. Across studies, darolutamide maintained a favorable safety profile, with adverse event rates similar to those of the placebo [81]. These advances reinforce ARPIs as essential components of modern prostate cancer care, giving patients more options for effective and well-tolerated treatments at both early and advanced stages of the disease [82].

AR splice variants, particularly AR-V7, play a critical role in the development of resistance to conventional AR-targeted therapies in prostate cancer. They lack the ligand-binding domain and remain constitutively active, thereby driving tumor progression despite androgen deprivation [16]. Therapeutic strategies targeting these variants include agents like niclosamide, which promotes degradation of AR-V7, and ASC-J9, which inhibits AR-V7 activity, both aiming to restore sensitivity to anti-androgen treatments [83,84]. Although these approaches show promise in preclinical and early clinical studies, they are still under clinical evaluation and have not yet been widely adopted in standard clinical practice [85].

DNA Damage Repair Pathway

**Figure 7:**
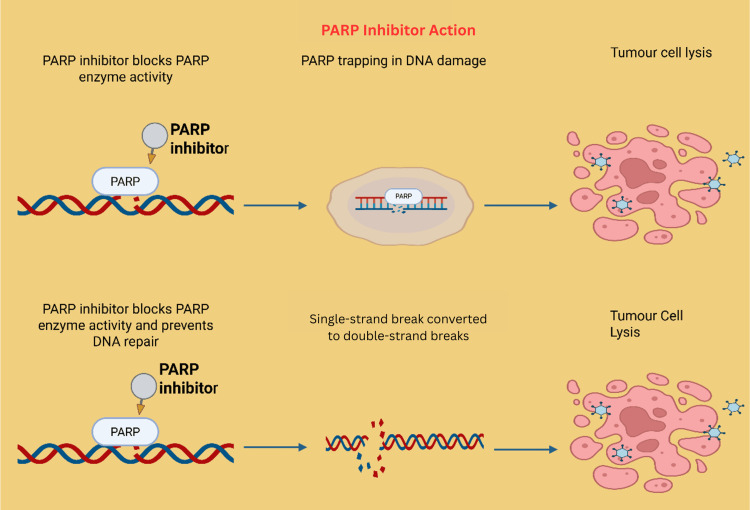
PARP inhibitor mechanism of action. By inhibiting the PARP protein, which normally repairs single-strand DNA breaks, PARP inhibitors prevent cancer cells from repairing their damaged DNA, ultimately leading to cell death Created using BioRender.com. PARP: poly (ADP-ribose) polymerase, DNA: deoxyribonucleic acid

Poly (ADP-ribose) polymerase (PARP) inhibitors represent a significant advancement in the treatment of mCRPC, particularly in patients harboring defects in homologous recombination repair (HRR) genes such as BRCA1 and BRCA2 [86]. Usually, DNA double-strand breaks and single-strand breaks occurring during the S phase of the cell cycle are accurately repaired through HRR, a process orchestrated by BRCA1/2. Tumor cells with deficient HRR rely heavily on the PARP1-mediated base excision repair pathway to maintain DNA integrity [87]. Inhibition of PARP enzymatic activity by PARP inhibitors leads to the accumulation of DNA damage, resulting in synthetic lethality and subsequent cancer cell death (Figure 7) [88]. Clinically, the FDA has approved olaparib, a PARP inhibitor, for use in mCRPC patients with deleterious or suspected deleterious HRR gene mutations who have progressed after treatment with novel ARPIs such as enzalutamide or abiraterone. This approval is supported by pivotal clinical trials, including the TOPARP-A study, which demonstrated that patients with BRCA1/2 mutations exhibited a markedly higher response rate to olaparib (14 of 16 patients) compared to those without such mutations (2 of 33 patients) [89]. The PROfound trial further corroborated these findings, showing that olaparib significantly improved radiologic progression-free survival (rPFS) and overall survival compared to standard therapies in patients with BRCA and ATM mutations [90]. In addition to olaparib, niraparib, a selective PARP-1/2 inhibitor, has gained regulatory approval in combination with abiraterone acetate (marketed as Akeega) for mCRPC patients with BRCA1/2 or other HRR mutations. The MAGNITUDE trial demonstrated that the combination of niraparib and abiraterone significantly delayed disease progression and improved rPFS compared to abiraterone alone, with the most significant benefit observed in the BRCA-mutated subgroup [91]. Nearly 50% reduction in disease progression risk was observed in patients with BRCA alterations [92]. These results underscore the expanding role of PARP inhibitors in precision oncology, offering targeted treatment options for genetically defined subsets of prostate cancer patients and highlighting the importance of molecular profiling in guiding therapeutic decisions.

Steroidogenesis Pathway Targeting

A key therapeutic strategy in advanced prostate cancer is inhibiting androgen production through the steroidogenesis pathway. Abiraterone acetate, an oral agent, acts as a selective and irreversible inhibitor of the CYP17A1 enzyme, which plays a critical role in the enzymatic conversion of steroid precursors such as pregnenolone and progesterone within the testes, adrenal glands, and tumor microenvironment [93]. By blocking both the 17α-hydroxylase and 17,20-lyase activities of CYP17A1, abiraterone markedly reduces the synthesis of testosterone and other androgens, including those produced inside the tumor, which are central drivers of CRPC progression [94].

Clinical trials have shown that abiraterone treatment leads to significant declines in prostate-specific antigen (PSA) levels, objective radiologic responses, and a reduction in circulating tumor cells, indicating that androgen signaling continues to sustain many cases of CRPC. In pivotal phase III studies, at least half the PSA levels dropped in nearly two-thirds of patients, with these responses often persisting for an extended period [93]. However, because CYP17 inhibition also interferes with cortisol production by inhibiting 17α-hydroxylase, a necessary step in the cortisol biosynthesis pathway, the body compensates by raising adrenocorticotropic hormone levels, which in turn increase mineralocorticoid production. This hormonal imbalance can prompt side effects like hypertension, hypokalemia, and fluid retention. Although low-dose corticosteroids such as prednisone are co-prescribed to counteract this excess, a notable proportion of patients still experience mineralocorticoid toxicity, with recent analyses reporting an approximate 15% increased risk even with steroid use [95,96].

Orteronel (TAK-700), another oral agent, selectively inhibits the 17,20-lyase function of CYP17, aiming to reduce androgen production while minimizing mineralocorticoid excess. While early studies showed durable declines in PSA and testosterone levels in CRPC, subsequent phase III trials failed to demonstrate improvements in overall survival. As a result, orteronel is not adopted as a standard treatment, and abiraterone remains central for targeting androgen synthesis in CRPC [97,98].

In conclusion, abiraterone is the established option for disrupting androgen synthesis in advanced prostate cancer. It provides significant clinical benefit, but ongoing monitoring for mineralocorticoid-mediated adverse events is essential. Other agents like orteronel have not matched abiraterone's efficacy and are not used in routine clinical practice.

PI3K/Akt/mTOR Pathway Inhibition

Loss of the PTEN gene is a common problem in CRPC. When PTEN is lost, the PI3K/Akt/mTOR pathway becomes overactive, helping cancer cells survive and making them resistant to standard treatments, such as drugs that block the AR [99]. Because these two pathways (AR and PI3K/Akt/mTOR) can compensate for each other, blocking only one often isn’t enough. Specifically, blocking the AR can increase the activity of the PI3K/Akt pathway (especially when PTEN is absent), and blocking the PI3K pathway can enhance AR signaling [100]. This is why researchers are testing strategies that block both paths simultaneously.

The most substantial evidence comes from a clinical trial called IPATential150, which examined the combination of ipatasertib (an AKT inhibitor) and abiraterone (an androgen biosynthesis inhibitor) in patients with cancer that lacked PTEN expression. Together, these drugs helped patients live longer without their cancer getting worse and reduced the risk of cancer progression by about 23%, but mainly in the patients who had lost PTEN [101]. Side effects such as rash, diarrhea, and high blood sugar did happen but were usually manageable [101].

Other drugs called PI3Kβ inhibitors, like GSK2636771, are also being studied in PTEN-deficient CRPC and may work even better when used with AR blockers [102]. Additionally, new AKT inhibitors, such as capivasertib, and drugs that block multiple points in the pathway simultaneously are in clinical trials; some of these may have fewer side effects or be more effective than ipatasertib [103,104]. Studies in the lab show that blocking AKT is especially good at stopping growth in prostate cancers missing PTEN, but over time, cancer can find ways to get around these drugs, and side effects are still a challenge [100,103].

Cytokine and Growth Factor Pathways

Recent evidence indicates that cytokines, particularly IL-6, can activate the STAT3 signaling pathway. This activation enhances AR activity even in the absence of androgens, a phenomenon known as ligand-independent activation. It contributes to resistance to standard therapies in prostate cancer, including castration-resistant and neuroendocrine variants [105,106]. Targeting this pathway has emerged as a promising strategy to overcome resistance, with preclinical studies showing that inhibitors of STAT3/JAK2, such as AG490, can reduce STAT3-mediated AR activation and hinder prostate cancer cell growth [107,108]. Additionally, receptor tyrosine kinases (RTKs), EGFR, HER2, and IL-6R/gp130-associated kinases like JAKs, which are involved in several growth factor signaling pathways, can also activate STAT3 and support tumor progression; hence, RTK inhibitors are actively being investigated alongside STAT3 inhibitors for their potential to block these resistance mechanisms and offer new treatment options for aggressive or neuroendocrine forms of prostate cancer [109,110].

Cell Cycle and Epigenetic Targeting

Cyclin-dependent kinases like CDK8 and CDK19, which regulate transcription via the mediator complex along with other proteins that control how genes are turned on or off, such as histone deacetylases (HDACs) and heat shock protein 90 (HSP90), are believed to play a role in how advanced prostate cancer (including CRPC) grows and becomes resistant to treatment. Researchers have found that these proteins can help cancer cells survive and adapt, making the disease more challenging to treat over time [111,112]. Because of this, new drugs designed to block CDK8/19, HDACs, or HSP90 are being studied in early clinical trials, especially for men whose cancer returns or stops responding to standard treatments [113,114]. Some early results are promising, showing that these drugs may slow cancer growth or help resensitize tumors to other therapies. However, larger studies are needed to determine which patients benefit most and to understand the risks and side effects well enough for regular clinical use [115].

Immune Checkpoint Inhibitors

**Figure 8:**
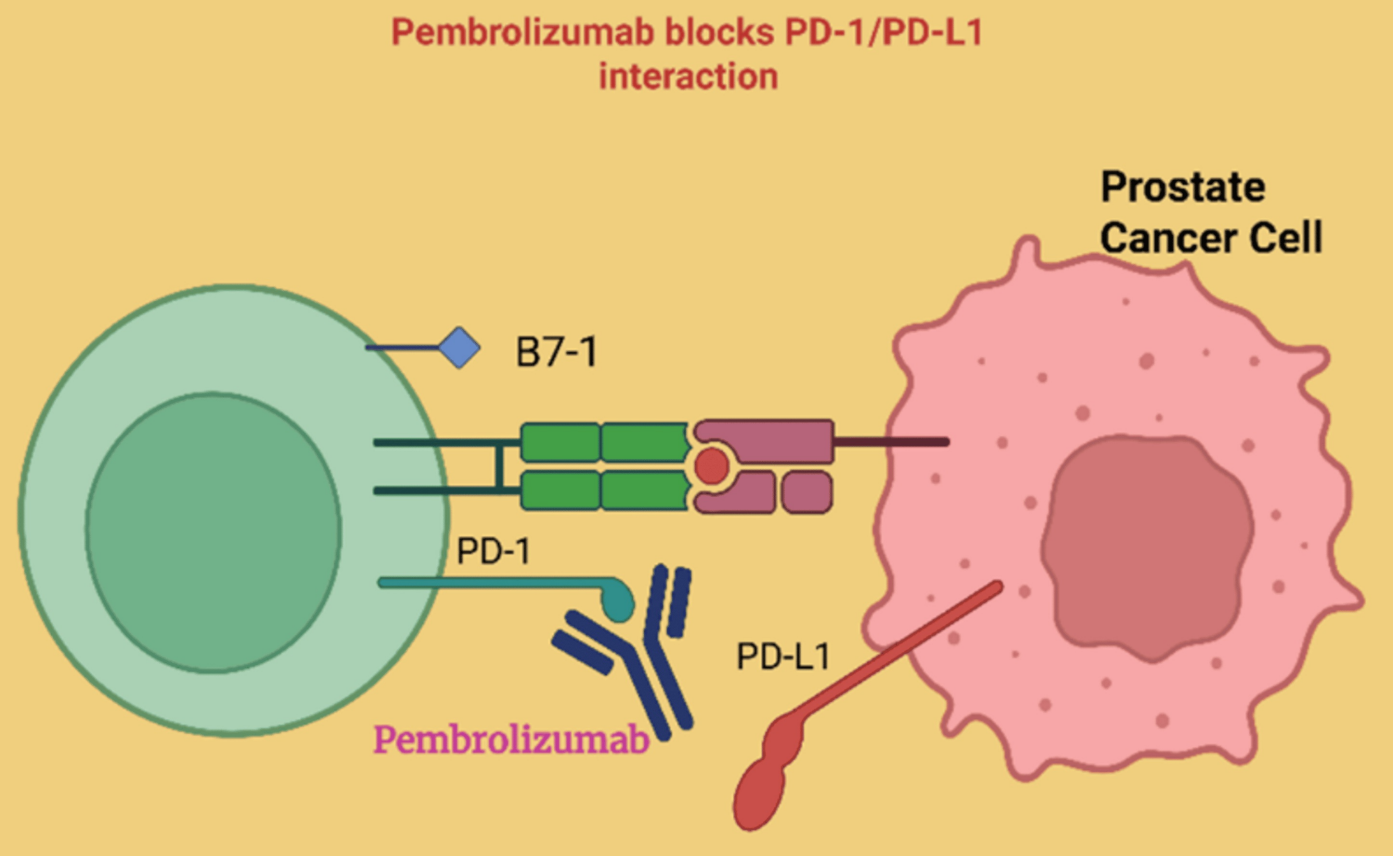
Pembrolizumab mechanism of action. Pembrolizumab binds to the PD-1 receptor on T-cells to prevent its interaction with PD-L1 on PROSTATE CANCER cells, thereby restoring T-cell activity and enabling an effective anti-tumor immune response Created using BioRender.com. PD-1: programmed cell death protein 1, PD-L1: programmed cell death ligand 1

Immune checkpoint inhibitor options for CRPC remain fairly limited as single treatments, but several agents are relevant, especially in certain biomarker-selected patient groups. Pembrolizumab, an anti-PD-1 antibody, is approved for CRPC only in tumors that are microsatellite instability-high (MSI-H) or mismatch repair-deficient (dMMR), which are rare in prostate cancer but represent a population with the best chance of benefit [115]. Pembrolizumab (Keytruda) works by blocking the interaction between PD-1 receptors on T-cells and PD-L1 on tumor cells, thereby unleashing the patient’s immune system to recognize and attack malignant cells (Figure 8) [116]. In the context of mCRPC, pembrolizumab is reserved for individuals with these molecular alterations, confirmed by validated molecular testing, because only a small subset of patients qualify for treatment. Clinical evidence, including results from Phase II/III trials and various case series, demonstrates that while the overall response rate is modest, those who do respond may experience a durable and clinically meaningful benefit [117]. In routine clinical practice, pembrolizumab remains available only for biomarker-positive cases, highlighting the importance of comprehensive tumor genomic profiling to identify eligible patients and optimize outcomes in advanced prostate cancer.

Other immune checkpoint inhibitors, such as nivolumab (anti-PD-1), atezolizumab (anti-PD-L1), and ipilimumab (anti-CTLA-4), have shown limited effectiveness as monotherapies in unselected CRPC populations; however, ongoing trials are evaluating them in various combination regimens [118]. Notably, the CONTACT-02 Phase III trial (2025) revealed improved progression-free survival when atezolizumab was combined with cabozantinib in mCRPC, supporting the value of combination strategies [119]. Emerging immunotherapies targeting novel molecules, such as B7-H3 (e.g., enoblituzumab, vobramitamab), are currently in clinical investigation, showing early evidence of immune activation [120]. Across all approaches, the most tremendous success with immune checkpoint inhibition is anticipated in biomarker-driven settings, particularly in patients whose tumors are MSI-H/dMMR, have high PD-L1 expression, CDK12 mutations, or elevated tumor mutational burden [121]. As a result, immune checkpoint therapy is best considered for select patients with these molecular features, often through well-designed clinical trials evaluating both monotherapy and combination approaches.

Tumor Vaccines

Sipuleucel-T (Provenge) is an autologous cellular immunotherapy and was the first FDA-approved cancer vaccine for mCRPC; it is primarily indicated for asymptomatic or minimally symptomatic patients, working by stimulating the patient’s immune system to target prostatic acid phosphatase (PAP), a prostate cancer-associated antigen [122]. The process involves harvesting peripheral blood mononuclear cells from the patient, incubating them with a recombinant PAP-GM-CSF fusion protein to activate antigen-presenting cells, and then reinfusing these cells into the patient [122,123]. The IMPACT Trial demonstrated a median overall survival benefit of approximately 4.1 months compared to the placebo (25.8 vs. 21.7 months), but little or no impact on measurable disease progression, PSA declines, or objective tumor shrinkage [122]. Beyond sipuleucel-T, other tumor vaccine strategies for CRPC, such as viral vector-based vaccines (e.g., PROSTVAC/PSA-TRICOM) and DNA or peptide vaccines, have focused on stimulating antigen-specific T-cell responses [124,125]. Although the vector-based vaccine PROSTVAC showed a median overall survival improvement of 8.5 months in a phase II trial, it failed to meet primary endpoints in the larger phase III PROSPECT trial, reflecting the ongoing challenges with cancer vaccines in prostate cancer [126]. Other peptide and DNA vaccines, including those targeting PAP, have generated robust immune responses and favorable safety profiles. However, definitive clinical benefits in terms of progression or overall survival are still being studied [127]. Notably, combination strategies, pairing vaccines with immune checkpoint inhibitors or androgen-deprivation therapy, are under investigation to boost antitumor efficacy [127]. In summary, sipuleucel-T has established proof of principle for immunotherapy, extending survival in mCRPC with modest benefits and minimal side effects. Continued research and ongoing trials aim to improve outcomes with tumor vaccines through more potent and integrative immunologic strategies [123,125].

Immunotherapy

**Figure 9:**
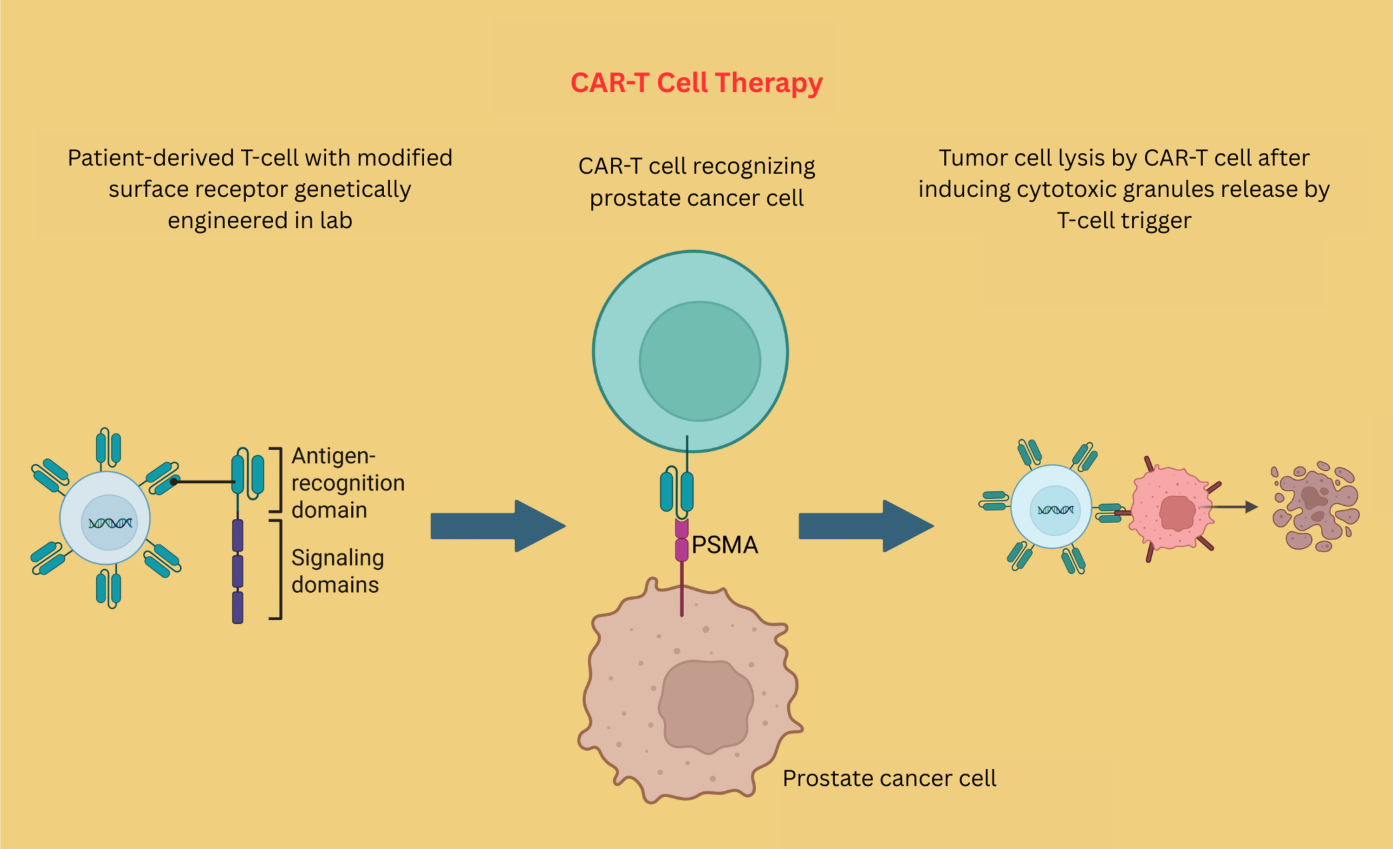
Immunotherapy in CRPC. CAR-T therapy involves engineering T-cells to recognize and target prostate cancer cells through T-cell activation Created using BioRender.com. PSMA: prostate-specific membrane antigen, CAR-T: chimeric antigen T-cell therapy

**Figure 10:**
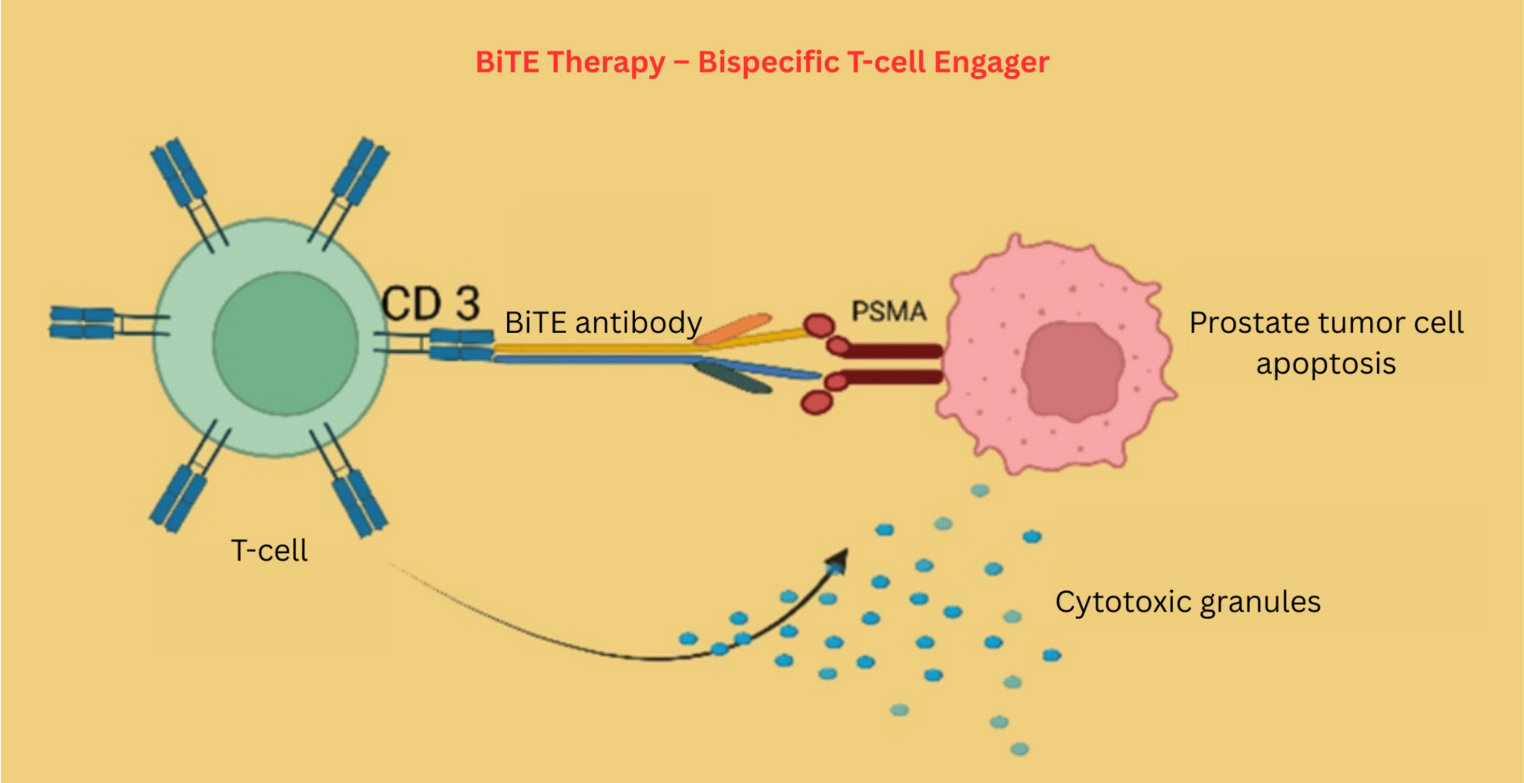
BiTE molecule acts as a bridge, simultaneously binding to a protein on a cancer cell and a T-cell, thereby bringing them into close proximity to trigger the T-cell to kill the cancer cell Created using BioRender.com. BiTE: bispecific T-cell engager, PSMA: prostate-specific membrane antigen

Chimeric antigen receptor T-cell (CAR-T) therapies and bispecific T-cell engagers (BiTEs) represent innovative immunotherapeutic approaches in the treatment of mCRPC. CAR-T therapies involve the genetic engineering of patients’ T-cells to express synthetic receptors, most commonly directed at PSMA, enabling precise targeting and killing of prostate cancer cells (Figure 9) [128]. Conversely, BiTEs are special antibody-based proteins that are created by linking two distinct antibody fragments. One part is designed to recognize a specific marker found on tumor cells, while the other part attaches to a particular part of T-cells called CD3. These two parts are joined together by a flexible link [129]. BiTEs facilitate direct T-cell activation by engaging CD3, thereby bypassing the need for classical costimulatory signals and enabling the cytotoxic killing of tumor cells (Figure 10) [130]. Although neither approach has gained regulatory approval for prostate cancer outside clinical trials, both are under active investigation in early-phase studies worldwide. Initial clinical trials suggest that trials of CAR-T and BiTE therapies may induce significant reductions in PSA levels and measurable tumor shrinkage in select patients with heavily pretreated or refractory mCRPC, with some cases demonstrating durable responses and disease control [131]. However, significant challenges remain, including modest and variable efficacy, the incidence of treatment-related toxicities such as cytokine release syndrome and neurotoxicity, and the complexity of manufacturing and delivering these cell-based or bispecific therapies [132]. Currently, these advanced immunotherapies are primarily utilized in clinical research settings, and no CAR-T or BiTE therapy has yet received FDA or EMA approval for prostate cancer. Still, ongoing trial results will further clarify their role and facilitate the development of safer, more effective strategies for advanced prostate cancer.

Combination Therapy

The combination of talazoparib, a PARP inhibitor, and enzalutamide, an ARPI, represents a significant advance in the management of mCRPC with HRR mutations [133]. Talazoparib disrupts cancer cell DNA repair mechanisms, while enzalutamide targets androgen signaling. Their dual action effectively interrupts two critical pathways that drive prostate cancer progression [134]. In 2023, this combination became the first FDA-approved regimen combining a PARP inhibitor with an ARPI for mCRPC in patients harboring HRR gene alterations, establishing a new treatment paradigm. The interim results of clinical evidence from the pivotal TALAPRO-2 study demonstrated that talazoparib plus enzalutamide significantly prolongs rPFS and overall survival compared to enzalutamide alone, with the effect being most pronounced among patients with BRCA mutations [135]. Notably, the safety profile was manageable, ensuring clinical practicality. As of 2023, this combination is available as a first-line therapy for individuals with HRR-mutated mCRPC, positioning it as the new standard of care for this patient population.

Role of exosomes in CRPC treatment

Exosomes are small vesicles secreted by cells, and they play a significant role in the progression of CRPC. Acting as carriers, they transfer various molecules such as proteins and nucleic acids between cells, thereby facilitating communication that supports tumor growth and survival. Exosomes help create a microenvironment that favors cancer development, promote angiogenesis to supply nutrients, and aid in immune evasion by carrying inhibitory molecules [136]. Moreover, they contribute to cancer metastasis by enhancing tumor cell mobility and invasiveness, and they are involved in the transfer of drug resistance proteins, leading to treatment challenges [136]. Additionally, since exosomes present in blood and urine often reflect the status of the tumor, they are being explored as potential noninvasive biomarkers for the diagnosis and monitoring of CRPC.

Exosome-based therapies for CRPC are currently under active investigation, with several experimental approaches showing encouraging results in preclinical studies. One notable strategy involves the use of engineered exosomes for targeted drug delivery. For instance, in 2024, researchers developed exosomes decorated with antibodies against CEACAM5, a marker found on NEPC cells, a subtype of CRPC. These exosomes were loaded with inhibitors for EZH2 and the AR, and studies in both cell cultures and animal models demonstrated significant tumor suppression, highlighting their potential as a therapeutic tool for CRPC [137].

Another promising approach is exosome-mediated delivery of microRNAs. Studies have used mesenchymal stem cell-derived exosomes to transport microRNA let-7c, a well-known tumor suppressor, into CRPC cells. This delivery inhibited tumor cell growth and migration in laboratory models, supporting the use of exosomes as vehicles for gene therapy in advanced prostate cancer [138].

Additionally, exosomes are being explored as carriers for immunotherapeutic agents and gene therapies [136]. Although current evidence from preclinical research is promising, most of these approaches are still at the experimental stage, and further study is required to establish their safety, targeting efficacy, and ability to address drug resistance in CRPC.

Currently, most exosome-based therapies for CRPC are still in early or preclinical development and have not yet progressed to advanced clinical trials or become standard patient care. Ongoing research is focused on improving the methods for exosome isolation, targeting, and drug loading to facilitate future clinical applications.

Importance of personalised treatment in CRPC

Personalized treatment has become very important in managing CRPC, especially as researchers learn more about how complex the disease is. By identifying precise changes in a patient’s cancer, such as issues with DNA repair, specific proteins like HSF1, ERs, and TOPK, or mechanisms by which the cancer evades the immune system, clinicians can select medications likely to be most effective for each individual. This represents a significant improvement over older methods, where the same treatment was administered to everyone. Customizing therapy based on each patient’s tumor can improve results and reduce side effects. As this article explains, personalized treatment not only offers new hope to patients with few options left but also improves their chances of fighting the disease and leading a better quality of life.

Tables 1-3 highlight key molecular targets, approved and investigational therapies, and their clinical indications in CRPC, providing a concise reference for ongoing advances in the field.

**Table 1:** List of drugs for CRPC currently available in clinical use 177Lu: lutetium-177, ARPI: androgen receptor pathway inhibitor, CRPC: castration-resistant prostate cancer, mCRPC: metastatic CRPC, mHSPC: metastatic hormone-sensitive prostate cancer, PSMA: prostate-specific membrane antigen, PARP: poly (ADP-ribose) polymerase, EMA: European Medicines Agency, FDA: Food and Drug Administration, MSI-H: microsatellite instability-high, dMMR: mismatch repair-deficient

Drug name/combination	Molecular target/mechanism	Indicated patient population/notes
Enzalutamide	ARPI (2nd gen)	Non-mCRPC and mCRPC; before/after chemotherapy
Apalutamide	ARPI (2nd gen)	High-risk non-mCRPC, mHSPC
Darolutamide	ARPI (2nd gen)	Non-mCRPC, well-tolerated
Abiraterone acetate	Androgen biosynthesis inhibitor (CYP17 inhibitor)	mCRPC, with prednisone, chemo-pre/post
Olaparib (alone/combination with abiraterone)	PARP inhibitor (DNA repair)	mCRPC with BRCA1/BRCA2 mutations; new EMA approval for combination therapy
Pluvicto (177Lu vipivotide tetraxetan)	PSMA-targeted radioligand therapy	PSMA+ mCRPC after ARPI, before chemotherapy; FDA expanded approval in 2025
Pembrolizumab, nivolumab, ipilimumab	anti-PD-1, anti-CTLA-4	mCRPC patients with MSI-H or dMMR
Tumor vaccines (e.g., sipuleucel-T)	Antigen-specific immune activation	mCRPC, better in minimally symptomatic, slow-growing disease; approved: sipuleucel-T in the USA

**Table 2:** List of drugs for CRPC in clinical trials ARPI: androgen receptor pathway inhibitor, CRPC: castration-resistant prostate cancer, mCRPC: metastatic CRPC, HRR: homologous recombination repair, PARP: poly (ADP-ribose) polymerase, ADC: antibody-drug conjugate, PSMA: prostate-specific membrane antigen, STEAP1: six transmembrane epithelial antigen of prostate 1, DART: dual-affinity re-targeting, CTLA-4: cytotoxic T-lymphocyte antigen 4, PD-1: programmed cell death protein 1, CAR-T: chimeric antigen receptor T-cell, AKT: protein kinase B, mTOR: mammalian target of rapamycin, MET: mesenchymal-epithelial transition factor, VEGFR2: vascular endothelial growth factor receptor 2, PI3K: phosphatidylinositol 3-kinase, HSP90: heat shock protein 90, HDAC: histone deacetylase, CDK: cyclin-dependent kinase, PSCA: prostate stem cell antigen

Drug name/combination	Molecular target/mechanism	Indicated patient population/notes
Talazoparib + enzalutamide	PARP inhibitor + ARPI	mCRPC, especially with HRR gene mutations; new standard per TALAPRO-2 trial
Talzenna (talazoparib), monotherapy or combo	PARP inhibitor	mCRPC, HRR-mutated; improved outcomes; FDA/EMA label expansion in 2025
ABBV-969	Dual-targeting ADC (PSMA + STEAP1)	mCRPC, overexpressing both targets; phase I in 2025
177Lu-J591, 225Ac-J591	PSMA-targeting radionuclide drug conjugates	mCRPC in trials, including with immunotherapy
Lorigerlimab	DART—dual CTLA-4/PD-1 checkpoint blockade	mCRPC, particularly chemorefractory; early clinical trials
BPX-601	PSCA-targeted CAR-T cell immunotherapy	mCRPC, clinical trial; first-in-human engineered CAR-T
Capivasertib, AKTi-CAPltello-280	AKT1/2/3 kinase inhibitor	mCRPC, in combination with docetaxel; ongoing phase III
Everolimus	mTOR inhibitor	mCRPC, often with bicalutamide; phase II/III trials
Cabozantinib	MET + VEGFR2 kinase inhibitor	mCRPC, advanced/refractory; failed Phase III survival benefit but still under study
AMG386 (trebananib)	Angiopoietin pathway inhibitor	mCRPC, in combination; in trials
BKM120	PI3K inhibitor	mCRPC, with abiraterone; in trials
Itraconazole	Hedgehog/angiogenesis pathway inhibitor	CRPC patient’s refractory to standard therapy; in trials
Ipatasertib	AKT inhibitor (AKT1/2/3 isoforms)	mCRPC, including PI3K pathway-altered; often combined with abiraterone or ARPIs
HSP90 inhibitors	HSP90 inhibitors	Early clinical trials: advanced, relapsed, or resistant CRPC
HDAC inhibitors	HDAC inhibitors	Early clinical trials: advanced, refractory, or relapsed CRPC. Used for patients failing standard AR-targeted therapies
CDK8/19 inhibitors	Cyclin-dependent kinase 8/19 inhibitors	Early clinical trials: refractory, relapsed, or non-responsive CRPC. Focus on therapy-experienced, resistant cases

**Table 3:** List of drugs for CRPC in experimental stage CRPC: castration-resistant prostate cancer, mCRPC: metastatic CRPC, ER: estrogen receptor, SERM: selective estrogen receptor modulator, STEAP: six-transmembrane epithelial antigen of prostate, SSTR: somatostatin receptor, CAR-T: chimeric antigen receptor T-cell, PSMA: prostate-specific membrane antigen, PSCA: prostate stem cell antigen, BiTE: bispecific T-cell engager, EpCAM: epithelial cell adhesion molecule, JAK2: janus kinase 2, STAT3: signal transducer and activator of transcription 3, LXRA/LXRB: liver X receptor alpha/beta, HSF1: heat shock factor 1, ARSI: androgen receptor signaling inhibitor, TOPK: T-LAK cell-originated protein kinase, CYP17: cytochrome P450 17A1 (17,20-lyase)

Drug/class	Molecular target/mechanism	Indicated patient population	Status
SERM	ER antagonist/modulator	Used off-label or in trials for patients progressing on standard CRPC therapy; experimental combos with somatostatin analogs	Experimental
STEAP inhibitor	STEAP	Investigational for STEAP1-overexpressing mCRPC	Experimental
SSTR inhibitor (e.g., octreotide, lanreotide, pasireotide)	SSTRs (SSTR1-3, SSTR5)	Neuroendocrine or SSTR-positive CRPC; often combined with standard therapies	Experimental/clinical trial
CAR-T	PSMA, PSCA, other tumor surface antigens	mCRPC, especially heavily pretreated or chemotherapy-refractory patients	Experimental/clinical trial
BiTEs	PSMA, STEAP1, EpCAM (binds tumor and CD3 on T-cells)	mCRPC, often after failure of other therapies, target-positive tumors	Experimental/clinical trial
AG490	JAK2/STAT3 pathway inhibitor	Not yet in clinical use; preclinical or early-phase for CRPC, especially for STAT3-driven tumors	Experimental
LXR agonists/inhibitors	LXRA/LXRB (nuclear hormone receptors regulating cholesterol/lipid)	Early investigation in preclinical CRPC models for anti-cancer and anti-metastatic effects	Experimental/preclinical
NXP800	Heat shock factor 1 (HSF1) inhibitor	Advanced/metastatic, enzalutamide-resistant, ARSI-resistant CRPC. Targets AR signaling and growth in highly resistant forms	Experimental
Cilengitide	αvβ3, αvβ5 integrin inhibitor	Investigational: advanced, therapy-resistant CRPC	Studies ongoing; target subset not well defined
Abituzumab	αv integrin monoclonal antibody	Investigational: metastatic CRPC with confirmed bone lesions	Potential in slowing bone lesion progression; not standard of care
OTS-514	TOPK inhibitor	Investigational: CRPC with AR-V7 variant/expression, therapy-resistant disease	Potential utility in AR-signaling independent/variant-driven resistance
Orteronel (TAK-700)	CYP17 17,20-lyase inhibitor	Investigational: progressive, chemotherapy-naïve CRPC	Not standard of care; evaluated in early, progressive, non-responsive disease

## Conclusions

The treatment approach for CRPC is rapidly advancing, bringing new opportunities with direct impact on patient care. As discussed in this article, the focus has shifted from only targeting the AR to exploring multiple molecular pathways, such as DNA repair defects, immune checkpoints, and innovative proteins like HSF1, ERs, and TOPK. This development is especially encouraging for patients who have not responded to existing options. The addition of novel therapies, including radioligand treatments and newer immunotherapies such as BiTEs and CAR-T cells, is expanding the treatment options available to doctors. Importantly, these changes highlight the necessity of detailed molecular and genetic tests to tailor treatments according to each patient’s tumor profile, thereby maximizing benefits and reducing unnecessary side effects. Looking ahead, as more therapeutic targets are identified and precision medicine becomes routine, there is strong hope for improved survival rates and better quality of life for men with advanced prostate cancer, paving the way for truly customized care.
